# AAEM’S Response to the Yale PA “Residency Program”

**DOI:** 10.5811/westjem.2021.6.53137

**Published:** 2022-01-01

**Authors:** Lisa Moreno-Walton

**Affiliations:** The American Academy of Emergency Medicine, Milwaukee, WisconsinAAEM Resident and Student Association, Milwaukee, Wisconsin

Dear Editor:

We believe the letter to the editor by Tsyrulnik et al^1^ clarifying the initial manuscript “Implementation of a Physician Assistant Emergency Medicine Residency Within a Physician Residency” from December 2020 is an important marker and acknowledgment of a deep-rooted workforce issue that will plague emergency medicine (EM) for the entirety of its future. It also only scratches the surface. Indeed, in the aftermath of the EM workforce reports by the American Academy of Emergency Medicine (AAEM), and more recently the American College of Emergency Physicians, the AAEM Resident and Student Association is now advocating for an end to all postgraduate training programs for non-physician practitioners (NPP).^2^

There is currently a logic paradox that threatens the quality of patient care in the United States. More than 25 states now allow nurse practitioners (NP) to care for patients without any physician involvement.^3^ This trend has now started for physician assistants (PA) as well and is rapidly gathering momentum in many state legislatures this year, coming on the heels of a global pandemic that saw many states relax supervision or collaboration regulations for both NPs and PAs.^4^

Both NPs and PAs have a fraction of the graduate medical education and training required for physicians to become independently licensed.^5^ Already, many patients with Emergency Severity Index scores 1 and 2 are seen independently by a PA or NP, and this is an important context in which the Yale PA graduate program is situated.^6^

Whether or not the authors intended any political inferences to be drawn from their study, *West*JEM readers and emergency physicians should be aware that they most certainly will be. The pressure on healthcare systems to do “more with less” is very real and a matter of survival for many. This will inevitably lead to consideration of replacing emergency physicians with NPPs, especially if they have postgraduate education that is inferred to be equivalent. To describe physician assistants as “independent providers of patient care,” as in the original manuscript, fuels the erroneous position that physicians and nonphysician practitioners are equivalent, and it was crucially important for the authors to clarify in their reply.

It is not fair to PAs and NPs to put them in the position of responsibility as independent practitioners because they do not have equivalent education and training to that of emergency physicians. The AAEM firmly believes that patients should have timely and unencumbered access to the most appropriate care led by a board-certified emergency physician.^7^

Additionally, words are of striking importance, and the language of a “Physician Assistant Emergency Medicine Residency” conflates a true residency for emergency physicians with that of additional, specific training for PAs who want to work in the EM environment. The AAEM, alongside multiple EM specialty organizations, opposes this language.^8^ Moreover, we do not support a PA or NP taking part in procedures that would take away from a physician resident’s education, nor do we support NPPs being trained in procedures that exceed the scope of their practice, such as procedural sedation, cricothyrotomy, and others.

The silver lining in the presumably unintentional ambiguous language of the original manuscript is that these issues have come to the forefront of conversation, and we appreciate the authors returning to the conversation to clarify that additional education and training short of the full, accredited education and training undertaken by physicians is insufficient for independent practice. The knowledge, skill sets, and hours of training for physicians are vastly different from those of NPs and PAs.^9^ Physician resident education must be the highest priority for any graduate training program to ensure the highest quality team leaders in the evolving EM work force.

We hope that academic and non-academic emergency departments alike take note of these issues and recognize the importance of physician-led patient care, as well as the threat to this care model that is being promulgated by the ongoing movement of NPPs to acquire independent practice in many states.

The safety of our patients is at stake.

Sincerely,

Lisa Moreno-Walton, MD, MS, President, The American Academy of Emergency Medicine (AAEM)

AAEM/RSA, AAEM Resident and Student Association (RSA)
